# Feedback activation of HER3 attenuates response to EGFR inhibitors in colon cancer cells

**DOI:** 10.18632/oncotarget.13834

**Published:** 2016-12-09

**Authors:** Albert Bosch-Vilaró, Bart Jacobs, Valentina Pomella, Layka Abbasi Asbagh, Richard Kirkland, Joe Michel, Sharat Singh, Xinjun Liu, Phillip Kim, Gregory Weitsman, Paul R. Barber, Borivoj Vojnovic, Tony Ng, Sabine Tejpar

**Affiliations:** ^1^ Laboratory of Molecular Digestive Oncology, Department of Oncology, KU Leuven, Leuven, Belgium; ^2^ Prometheus Laboratories, San Diego, CA, USA; ^3^ Richard Dimbleby Department of Cancer Research, Randall Division & Division of Cancer Studies, King's College London, Guy's Medical School Campus, London, UK; ^4^ Department of Oncology, Cancer Research UK and Medical Research Council Oxford Institute for Radiation Oncology, University of Oxford, Oxford, UK; ^5^ Breast Cancer Now Research Unit, King's College London, London, UK; ^6^ UCL Cancer Institute, Paul O'Gorman Building, University College London, London, UK

**Keywords:** colorectal cancer, cetuximab resistance, HER3, dimerization, feedback loop

## Abstract

The EGFR inhibitor cetuximab is approved for the treatment of colorectal cancer. However, both innate and acquired resistance mechanisms, including compensatory feedback loops, limit its efficacy. Nevertheless, the emergence of these feedback loops has remained largely unexplored to date. Here, we showed feedback upregulation of HER3 and induction of HER3 phosphorylation after cetuximab treatment in colon cancer cells. We also showed that this upregulation occurs, at least partly, through AKT inhibition. Together with this, we observed increased HER2:HER3 dimerization upon cetuximab treatment. Interestingly, lapatinib, a dual EGFR and HER2 tyrosine kinase inhibitor, blocked the increase of cetuximab-induced HER3 phosphorylation. Additionally, we showed that upon HER3 knockdown, cetuximab combined with lapatinib was able to decrease cell viability compared to HER3 expressing cells. These results suggest the existence of a cetuximab-induced feedback HER3 activation that could potentially result in reduced cetuximab efficacy in colorectal cancer patients. Taken together, we provide evidence of the limited effectiveness of cetuximab monotherapy compared to rational combinations.

## INTRODUCTION

The development of targeted anti-cancer agents has revolutionized cancer therapy during the last decades. For the treatment of colorectal cancer, antibodies targeting the epidermal growth factor receptor (EGFR) like cetuximab and panitumumab were the first molecularly targeted therapeutics to enter the clinical arena [1–3] and they are approved as first line treatment for *RAS* wild-type non-resectable metastatic colorectal cancer [4–7].

Although responses in an unselected patient population were dismal, the development of positive and negative predictive biomarkers has led to significantly improved patient selection and has resulted in increased clinical benefit [8]. While the use of positive predicting factors indicating EGFR activation such as EGFR ligand expression still warrants prospective validation [9–11], the use of negative predictive oncogenic mutations in *KRAS*, *BRAF*, *NRAS* and *PIK3CA* exon 20 as stratification factors resulted in nearly a doubling of the response rate to 41% of quadruple wild-type tumors in the first line setting [12]. Although they are prevalent in a lower proportion of tumors, additional gene amplifications of *HER2* and *MET*, among other alterations, were previously linked to cetuximab resistance [13, 14]. Recently, *HER2*, *EGFR, PDGFRA* and *MAP2K1* mutations and *FGFR1* amplifications have been identified as plausible innate resistance mechanisms to EGFR targeted therapy in colorectal cancer [15], as well as *ALK*, *FGFR2*, *NTRK1/2* and *RET* kinase addition [16].

Clinically, when treated with cetuximab, *KRAS* wild-type colorectal cancers experience on average a tumor decrease of only 41% after 24 weeks of treatment, leaving a significant tumor mass that could potentially develop additional genetic variant alleles associated with resistance. This includes *EGFR* acquired ectodomain mutations [17] and *KRAS* mutations [18, 19] among others. These reports advocate the need for therapeutic regimens achieving superior tumor kill. These could not only improve outcome through larger tumor shrinkage, but potentially prevent or delay the emergence of acquired resistance and disease progression.

From a biological standpoint, a body of evidence across tumor types suggests that antibodies targeting ErbB family members mainly function by blocking downstream signaling, which are prone to be compensated for by parallel signaling. Several compensatory feedback loops have been recently identified in the context of BRAF inhibitors [20, 21], phosphatidylinositol 3-kinase (PI3K)/AKT inhibitors [22] and MEK inhibitors [23] among others, leading to acquired drug resistance. This diverse array of resistance mechanisms seems to converge on feedback activation of the PI3K/AKT and the mitogen-activated protein kinase (MAPK)/extracellular-signal-regulated kinase (ERK) pathways, ultimately priming cancer cells for resistance [24]. Although ErbB inhibitors were the first targeted agents developed for the treatment of colorectal cancer, the emergence of these feedback loops has remained largely unexplored to date in the context of these drugs.

In this study, we aimed to determine whether feedback signaling induced by EGFR inhibitors could contribute to a decreased efficacy of EGFR inhibitors in colon cancer cells. Here, we showed that upon cetuximab treatment feedback induction of HER3 phosphorylation occurs, which is at least partly a result of AKT inhibition. This is coupled with a dimerization shift towards HER2:HER3 heterodimers. We also provided evidence that the dual EGFR/HER2 inhibitor lapatinib can prevent the cetuximab-induced HER3 phosphorylation and decrease cell viability.

## RESULTS

### Feedback induction of HER3 phosphorylation occurs after EGFR inhibition with cetuximab

**Figure 1:**
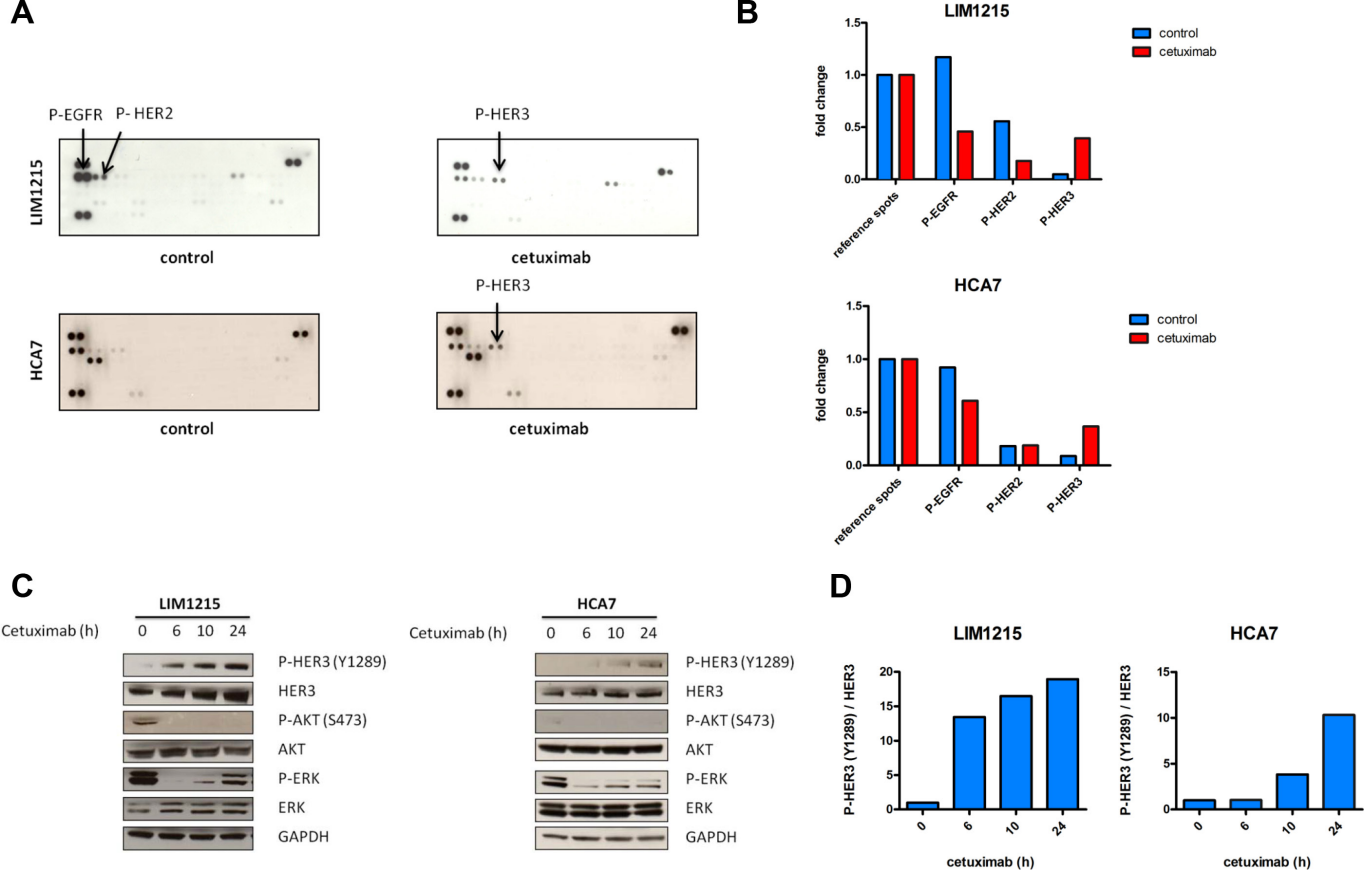
Feedback induction of HER3 phosphorylation after 24h EGFR inhibition with cetuximab (**A**) LIM1215 and HCA7 cells were treated for 24h with cetuximab (50 μg/mL) or control-treated with DMSO and phosphorylation of a set of RTKs was assessed with RTK arrays as described in the Materials and Methods section. (**B**) RTK arrays quantification. (**C**) LIM1215 and HCA7 cells were treated with cetuximab (50 μg/mL) for different time points. Protein levels were detected by western blotting as described in the Materials and Methods section. (**D**) Western blotting quantification of P-HER3/HER3.

Several reports have shown feedback activation of Receptor Tyrosine Kinases (RTK) upon inhibition of different components of the EGFR pathway [20–23]. Here, we wanted to identify RTKs that were potentially activated during treatment with the monoclonal antibody EGFR inhibitor cetuximab in previously identified intermediate cetuximab-sensitive *KRAS* and *BRAF* wild-type colon cancer cell lines. For this purpose, we utilized phosphorylated RTK arrays with LIM1215 and HCA7 cells (Figure 1A) [16, 18, 25]. As expected, EGFR phosphorylation was found significantly inhibited during 24h cetuximab treatment compared with control treatment in both cell lines. Additionally, a clear induction of HER3 phosphorylation was observed upon treatment, also in both cell lines.

We next assessed whether the observed cetuximab-induced feedback activation of HER3 was time-dependent and whether components downstream of EGFR were also altered. We hypothesized that cetuximab sensitivity relies mainly on the combined inhibition of the PI3K/AKT and MAPK/ERK signaling pathways. In order to identify potential compensatory activation of these pathways, we performed a temporal analysis of the LIM1215 cell line treated with cetuximab. Western blotting analysis in LIM1215 cells revealed that after initial inhibition of ERK and AKT phosphorylation, rebound activation of ERK occurred from 10h onwards (Figure 1C). Concurrent with the initial inhibition of ERK and AKT phosphorylation after 6h cetuximab treatment, HER3 protein levels increased (Figure 1C). This was coupled with a parallel activation of HER3 at one of its 6 PI3K p85 binding sites, Tyr1289, which becomes more evident after prolonged cetuximab treatment (Figure 1C and 1D). We confirmed these results in an additional cell line, HCA7 (Figure 1C and 1D). Together, this suggests that, upon cetuximab treatment, feedback HER3 activation may occur, which could potentially be associated with ERK and AKT inhibition.

### AKT inhibition drives HER3 compensatory feedback upregulation

**Figure 2:**
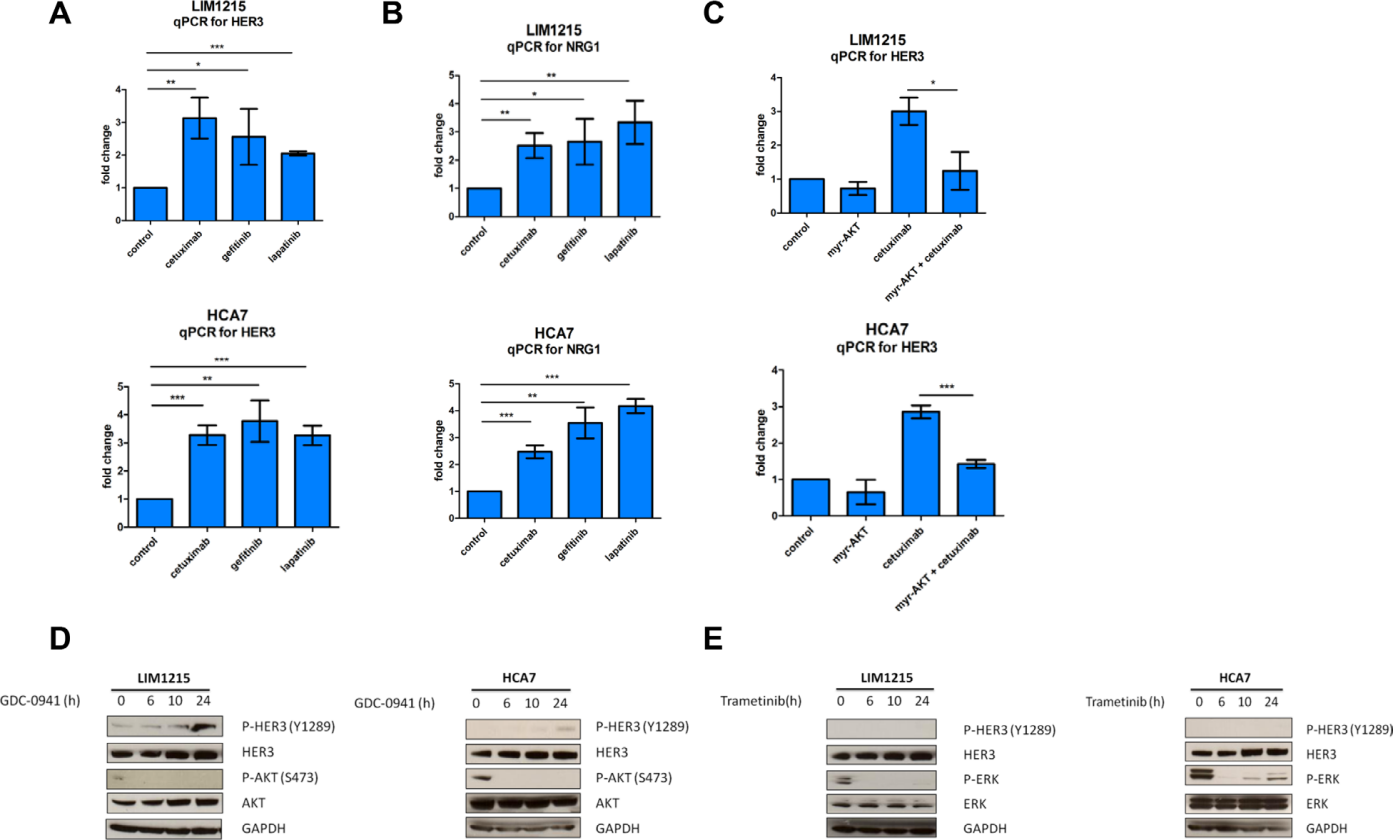
HER3 mRNA induction by cetuximab is blocked by AKT overexpression (**A** and **B**). LIM1215 and HCA7 cells were treated with cetuximab (50 μg/mL), gefitinib (1 μM), lapatinib (1 μM) or control-treated with DMSO for 24h (*n* = 3) and HER3 and NRG1 expression were measured by qPCR as described in the Materials and Methods section. Student's *t*-test significance: *p < 0.05, **p < 0.01, ***p < 0.001. (**C**) Transient overexpression of the AKT constitutively active mutant myristoylated AKT (myr-AKT) in LIM1215 and HCA7 cells. Cells were treated with cetuximab (50 μg/mL) or control-treated with DMSO for 24h (*n* = 3) and HER3 expression was measured by qPCR as described in the Materials and Methods section. Student's *t*-test significance: *p < 0.05, **p < 0.01, ***p < 0.001. (**D**) LIM1215 and HCA7 cells were treated with GDC-0941 (1 μM) or (**E**) trametinib (0.1 μM) for different time points. Protein levels were detected by western blotting as described in the Materials and Methods section.

Subsequently, we investigated which mechanisms downstream of EGFR could result in feedback activation of HER3 upon cetuximab treatment. Multiple reports in other cancer types indicate that feedback induction of RTKs occurs through relief of negative feedback signaling, such as AKT-regulated, FOXO-mediated HER2 and HER3 transcription [26, 27]. First, we assessed if inhibiting EGFR would also result in HER3 mRNA upregulation as a result of negative feedback signaling disruption. To test this, we compared HER3 mRNA levels in LIM1215 and HCA7 cells treated with different EGFR inhibitors including cetuximab, the EGFR tyrosine kinase inhibitor gefitinib and the dual EGFR and HER2 tyrosine kinase inhibitor lapatinib. Here, we observed that all three drugs induced a significant compensatory mRNA upregulation of HER3 within 24h (Figure 2A). Concomitant to this, we also observed an increase in the HER3 ligand heregulin (NRG1) mRNA levels after cetuximab, gefitinib and lapatinib treatments (Figure 2B). Next, we investigated if the observed mRNA upregulation of HER3 is due to relief of negative feedback exercised by AKT, as described in other cancer types. For this purpose, we performed transient overexpression of myristoylated AKT, an AKT constitutively active mutant construct [28]. Overexpression of myristoylated AKT in LIM1215 cells repressed HER3 transcription by nearly 2-fold in basal conditions, and was able to significantly prevent the transcriptional changes in HER3 induced by cetuximab in these cells (Figure 2C). This was also confirmed in HCA7 cells (Figure 2C). These data are in line with previous reports in the context of breast cancer [26] and suggest that AKT signaling downstream of EGFR represses the transcription of HER3 in basal conditions. Consequently, EGFR inhibition would block AKT and thus allow upregulation of HER3 mRNA. This was further supported by western blotting temporal analyses in LIM1215 and HCA7 cells where we showed that inhibiting PI3K, which is situated upstream of AKT, with GDC-0941 leads to induction of HER3 phosphorylation (Figure 2D). Next, we wanted to investigate if the MAPK/ERK signaling pathway also contributes to the observed HER3 compensatory feedback upregulation. To test this, we performed a time course of inhibition of the downstream modulator of the MAPK/ERK signaling pathway MEK with the tyrosine kinase inhibitor trametinib. Here, we showed that blocking the MAPK/ERK pathway with trametinib does not lead to HER3 phosphorylation induction (Figure 2E). Taken together, these results suggest that feedback activation of HER3 occurs, at least partly, through AKT inhibition.

### Cetuximab induces a dimerization shift towards HER2:HER3 heterodimers

**Figure 3:**
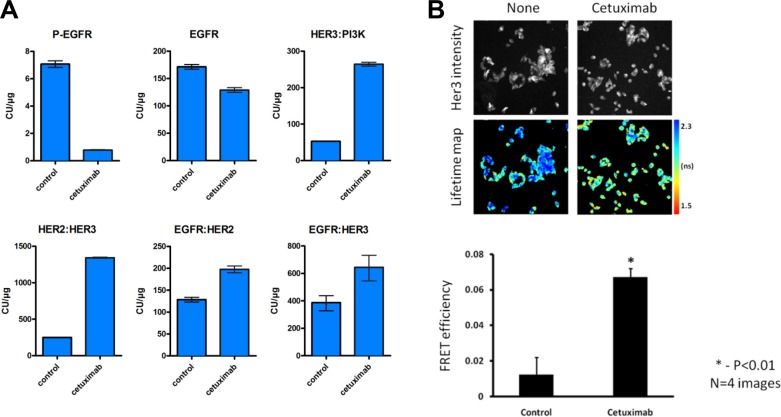
Cetuximab results in increased heterodimer formation and HER3:PI3K binding (**A**) LIM1215 cells were treated with cetuximab (50 μg/mL) for 24h and heterodimer formation between EGFR family members as well as both total and phosphorylated levels of EGFR were assessed by CEER by Prometheus Laboratories Inc. San Diego, CA (USA). The results are expressed as mean ± SEM. A total of 6 microarray spots were analyzed for each sample. CU: computed unit. (**B**) Dimerization of HER2:HER3 proteins induced by cetuximab demonstrated by FRET/FLIM analysis. LIM1215 cells were seeded on glass coverslips. 2 days later media was changed to serum free for 24h prior treatment with cetuximab (100 nM, 4h). Cells were fixed and stained with anti-HER3-IgG-Alexa546 (donor) and anti-HER2-IgG-Cy5 (acceptor) antibodies. Lifetime images acquired on custom build microscope and analyzed with TRI2 software [44]. The difference between untreated and treated cells is statistically significant (data are expressed as means ± SEM, *−*P* < 0.01, Student's *t*-test) (*n* = 4).

Our results showed that feedback HER3 activation occurs in LIM1215 and HCA7 colon cancer cells upon cetuximab treatment (Figure 1). However, HER3 has an impaired kinase activity and requires other partners to gain signaling capacity [29]. ErbB receptors cannot only form homodimers but also heterodimers with other family members. Hence, we explored whether the cetuximab-induced HER3 phosphorylation could be attributed to increased dimerization between ErbB family members. To this end, we utilized a highly sensitive proximity-mediated immunoassay, Collaborative Enzyme Enhanced Reactive-immunoassay (CEER) to quantify EGFR:HER2, EGFR:HER3 and HER2:HER3 heterodimer levels, HER3:PI3K p85 binding and receptor phosphorylation in a multiplexed array format using LIM1215 cell lysates [30]. These exploratory experiments showed that EGFR phosphorylation was clearly inhibited upon 24h of cetuximab treatment (Figure 3A), in line with our previous RTK array result (Figure 1A and 1B). Moreover, we observed a simultaneous 5.4-fold induction of HER2:HER3 dimers, which was associated with a 5-fold increase in HER3:PI3K p85 binding and a 1.3-fold decrease in EGFR protein levels (Figure 3A). Additionally, our exploratory experiments showed a decrease in HER2 phosphorylation and an increase in HER3 phosphorylation after cetuximab treatment (Supplementary Figure S1), in agreement with our RTK array result (Figure 1A and 1B). These data provide more evidence concerning feedback HER3 activation upon cetuximab treatment suggested by RTK arrays and western blotting (Figure 1). Moreover, they advocate that heterodimerization between HER2 and HER3 is increased in LIM1215 cells upon cetuximab treatment.

Next, we were interested in confirming the existence of HER2:HER3 dimers upon cetuximab treatment previously determined by CEER exploratory experiments in LIM1215 cells. We utilized a validated, FFPE-compatible fluorescence lifetime imaging microscopy (FLIM)-based assay [31–35], which is an accepted gold standard technique for measuring protein-protein interactions typically within the < 10 nm range (between the centres of the donor and acceptor fluorophores, that are directly conjugated to the antibodies, see Materials and Methods section), to quantify the *in situ* interaction between HER2 and HER3 in LIM1215 cells. We found increased HER2:HER3 dimerization after cetuximab treatment in LIM1215 cells (Figure 3B), as previously identified by CEER method (Figure 3A). The pseudocolor lifetime map for cells treated with cetuximab shows a decrease in fluorescence lifetime (expressed in ns) compared to untreated cells, which represents energy transfer from the donor to acceptor fluorophore due to the nanoscale proximity of both fluorophores. FRET efficiency, which is the readout of the fraction of HER3 bound to HER2, was significantly increased upon cetuximab treatment compared to control treatment. Taken together, these data show induction of HER2:HER3 dimers after cetuximab treatment in LIM1215 cells by means of both CEER and FRET/FLIM techniques.

### Inhibition of HER3 activation through multi-ErbB combination regimens

**Figure 4:**
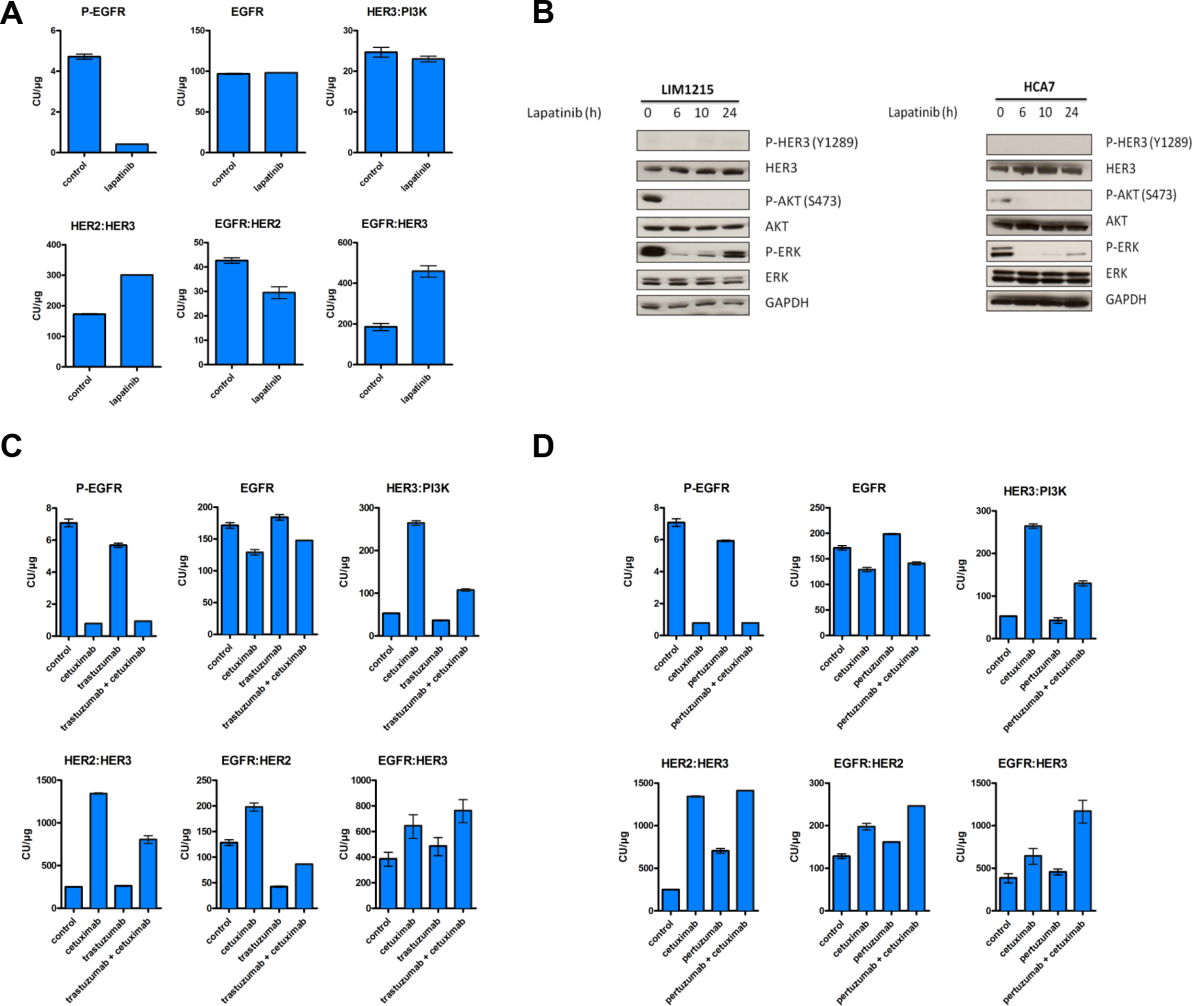
Time-dependent increase in HER3 phosphorylation does not occur upon lapatinib treatment LIM1215 cells were treated with (**A**) lapatinib (1 μM), (**C**) cetuximab (50 μg/mL) and trastuzumab (20 μg/mL) or (**D**) cetuximab (50 μg/mL) and pertuzumab (10 μg/mL) for 24h and heterodimer formation between EGFR family members as well as both total and phosphorylated levels of EGFR were assessed by CEER by Prometheus Laboratories Inc. San Diego, CA (USA). The results are expressed as mean ± SEM. A total of 6 microarray spots were analyzed for each sample. CU: computed unit. (**B**) LIM1215 and HCA7 cells were treated with lapatinib (1 μM) for different time points. Protein levels were detected by western blotting as described in the Materials and Methods section.

HER3 phosphorylation can be mediated by multiple kinases such as Src [36], MET [37] and FGFR2 [38] in addition to its family members EGFR and HER2. Since our RTK arrays did not reveal high phosphorylation levels of neither MET nor FGFR2 in LIM1215 cells after cetuximab treatment (Figure 1A), we hypothesized that HER3 is predominantly phosphorylated by EGFR or HER2, which has decreased levels of activation (Figure 1), but still can phosphorylate HER3 due to increased dimerization with it (Figure 3). Hence, we sought to identify whether combination strategies using EGFR and HER2 targeted drugs could inhibit the previously observed cetuximab-induced feedback activation of HER3. First, we evaluated whether monotherapy with the EGFR kinase inhibitor gefitinib and the reversible EGFR/HER2 kinase inhibitor lapatinib could prevent the cetuximab-induced activation of HER3 in LIM1215 cells. In exploratory experiments using CEER, treatment with gefitinib showed results in the same line as cetuximab treatment in terms of induction of heterodimer formation, HER3 protein levels and phosphorylation and elevation of HER3:PI3K p85 binding (Supplementary Figure S2B). However, treatment with he combined EGFR/HER2 kinase inhibitor lapatinib showed a blockage in the increase in HER3 phosphorylation and HER3:PI3K p85 binding (Figure 4A and Supplementary Figure S2A). It is thus interesting that even if lapatinib treatment increased NRG1 mRNA levels and HER3 mRNA and protein levels compared to control treatment (Figure 2A and 2B and Supplementary Figure S2A), it normalized both HER3 phosphorylation and associated HER3:PI3K p85 binding. This suggests that HER2-HER3 crosstalk is required to achieve feedback upregulation of HER3 together with receptor phosphorylation. The effect of lapatinib on preventing the increase in HER3 phosphorylation observed upon cetuximab treatment was further supported by a western blotting temporal analysis (Figure 4B). Additionally, while cetuximab treatment lead to a 5.4-fold induction of HER2:HER3 heterodimers, lapatinib treatment only showed a 1.7-fold increase (Figure 3A and Figure 4A). Moreover, both gefitinib and lapatinib treatments showed a decrease in ERK and AKT phosphorylation compared to control treatment. Concerning ERK, a 4.7-fold and a 5.5-fold decrease were observed respectively and for AKT a 2.4-fold and a 7.1-fold, suggesting that lapatinib inhibits ERK and AKT phosphorylation more potently than gefitinib (Supplementary Figure S2A and S2B).

Next, using the same rationale, we tested different drug combinations targeting EGFR and HER2. We treated LIM1215 cells combining cetuximab with the monoclonal antibody HER2 inhibitor trastuzumab and cetuximab with the HER2 dimerization inhibitor pertuzumab to find drug combinations that could alter the cetuximab-induced feedback activation of HER3. Co-treatment with cetuximab and trastuzumab could partially inhibit EGFR:HER2 dimer formation (Figure 4C). In contrast, we observed no decrease in HER3 phosphorylation, potentially due to the remaining EGFR:HER3 dimer levels that we observe in this context (Supplementary Figure S2C and Figure 4C). Co-treatment with cetuximab and pertuzumab did not inhibit heterodimer formation nor prevented HER3 phosphorylation (Figure 4D and Supplementary Figure S2D). Both co-treatments showed an induction of total levels of HER3 compared to baseline conditions (Supplementary Figure S2C and S2D). In terms of ERK and AKT, both co-treatment with cetuximab and trastuzumab and co-treatment with cetuximab and pertuzumab reduced the phosphorylated levels of both molecules compared to control treatments (Supplementary Figure S2C and S2D). The reduction on AKT phosphorylation upon these two co-treatments and upon gefitinib and lapatinib monotherapy, together with the induction of total levels of HER3 in all these treatments, is in line with our previous results where we showed that feedback activation of HER3 occurs, at least partly, through AKT inhibition.

Taken together, we showed that lapatinib is able to block the increase in cetuximab-induced HER3 phosphorylation, suggesting that HER2 facilitates HER3 signaling.

### HER3 inhibition increases the sensitivity of cancer cells to ErbB inhibitors

**Figure 5:**
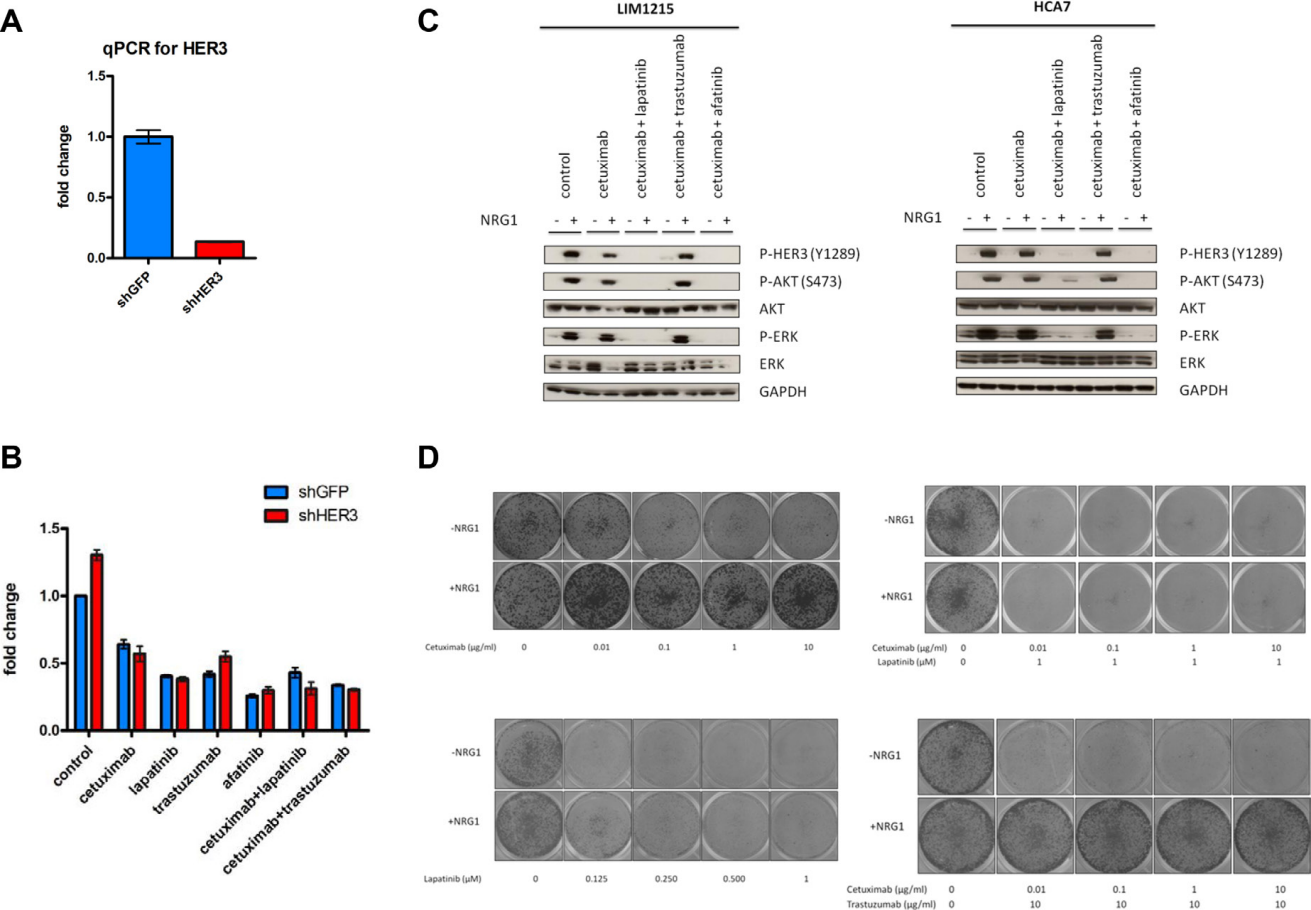
HER3 knockdown sensitizes LIM1215 cells to EGFR inhibitors and co-treatment of cetuximab with lapatinib prevents HER3 activation and resistance through NRG1 stimulation (**A**) Validation of HER3 knockdown in LIM1215 cells. HER3 expression was measured by qPCR as described in the Materials and Methods section. (**B**) LIM1215 cell viability measurement upon shRNA-based genetic inhibition of HER3 (shHER3) or control (shGFP) after 72h treatment with different drugs as described: control-treatment with DMSO, cetuximab (50 μg/mL), lapatinib (1 μM), afatinib (1 μM) and trastuzumab (20 μg/mL). Data are expressed as mean ± standard deviation. (**C**) LIM1215 and HCA7 cells were treated as described with the following drugs: control-treated with DMSO, cetuximab (50 μg/mL), lapatinib (1 μM), trastuzumab (20 μg/mL) or afatinib (1 μM). Cells were treated for 2h in the presence or absence of 10 min NRG1 stimulation (50 ng/mL). Protein levels were detected by western blotting as described in the Materials and Methods section. (**D**) LIM1215 10-day colony formation upon treatment with different drug combinations as described and in the presence or absence of NRG1 (50 ng/mL).

We observed that, after different EGFR and HER2 inhibition strategies, upregulation of HER3 and incomplete inhibition of AKT and ERK occurred (Figure 1, Supplementary Figures S1 and S2). Therefore, we hypothesized that inhibition of HER3 could lead to an increased sensitivity to ErbB inhibitors. To test this, we utilized a shRNA-based genetic inhibition strategy and assessed monotherapy and combination strategies of EGFR and HER2 inhibitors in LIM1215 cells, based on our results showing the ability of lapatinib to block the increase in HER3 phosphorylation (Figure 4B and Supplementary Figure S2A). Notably, treatment of cetuximab combined with lapatinib was already more potent than cetuximab treatment alone and knockdown of HER3 could additionally sensitize LIM1215 cells to this combination (Figure 5B). Due to the observed ability of lapatinib to prevent an increase in HER3 phosphorylation (Figure 4B and Supplementary Figure S2A), we tested an additional EGFR/HER2 inhibitor, namely the irreversible covalent EGFR/HER2 tyrosine kinase inhibitor afatinib. The effect observed upon lapatinib treatment was different for afatinib, which resulted in a more potent inhibition and which remains unexplained. Indeed, treatment with afatinib resulted in a decrease in cell viability compared to lapatinib, while these cells were not further sensitized to afatinib by knockdown of HER3.

Next, we wanted to provide further evidence on the need of HER2-HER3 crosstalk for the feedback HER3 phosphorylation presented before by altering the activity of HER3. To this end, we activated HER3 in LIM1215 cells through exposure to the exogenous HER3 ligand NRG1 and tested short-term treatment combinations with EGFR and HER2 inhibitors. As expected, HER3, AKT and ERK phosphorylation were potently induced in the presence of NRG1 (Figure 5C). Cetuximab was not able to inhibit this induction of HER3, AKT and ERK phosphorylation. Similarly, resistance to cetuximab was induced by treatment of these cells with NRG1 when measured in a long-term proliferation assay (Figure 5D). However, the observed NRG1-induced HER3, AKT and ERK phosphorylation could be prevented by co-treatment of cetuximab with lapatinib or afatinib (Figure 5C). This was confirmed in HCA7 cells (Figure 5C). Additionally, co-treatment with cetuximab and lapatinib was found capable to revert the NRG1-induced resistance to cetuximab in LIM1215 cells in a long-term proliferation assay (Figure 5D). Together, these data support the HER2-HER3 crosstalk required for endogenous feedback HER3 phosphorylation upon cetuximab treatment and suggests that co-treatment with cetuximab and lapatinib may warrant further investigation in order to prevent therapy resistance.

## DISCUSSION

Cetuximab is approved for the treatment of metastatic colorectal cancer but its response rate in *KRAS*, *BRAF*, *NRAS* and *PIK3CA* exon 20 quadruple wild-type tumors accounts for only 41%, leaving a high percentage of non-responders [12]. Furthermore, both innate and acquired resistance mechanisms previously described in this manuscript limit cetuximab's effectiveness [13–19]. Moreover, there is evidence of resistance through feedback loops in the context of different targeted drugs including BRAF, PI3K/AKT and MEK inhibitors [20–23]. These evidences point towards a rather low success rate of monotherapy targeted anticancer agents for the treatment of colorectal cancer.

Here, we showed the occurrence of a cetuximab-induced time-dependant increase in total HER3 protein levels and HER3 phosphorylation in *KRAS* and *BRAF* wild-type cells (Figure 1 and Supplementary Figure S1). Time-dependant feedback HER3 activation after EGFR inhibition has also been proposed in the context of breast cancer, although these breast cancer cells already presented with HER3 phosphorylation at baseline [39].

Moreover, we also showed that mRNA upregulation of HER3 occurred within 24h of cetuximab, gefitinib and lapatinib treatment (Figure 2A), suggesting that HER3 expression is negatively regulated by signaling downstream of EGFR. AKT-regulated feedback induction of RTKs through relief of negative feedback signaling has been previously shown [26, 27]. Furthermore, it has been described that the PI3K/AKT pathway is mainly mediated through HER3 [40, 41]. Using an AKT constitutively active mutant construct, we showed that the transcriptional changes in HER3 induced by cetuximab could be prevented (Figure 2C). This suggests that feedback activation of HER3 occurs, at least partly, through AKT inhibition, although we were not able to elucidate through which exact mechanism this occurs. This is in line with previous reports in breast cancer cells where myristoylated AKT prevents the escape of HER3 to EGFR inhibition with gefitinib treatment [39]. Concomitant to this, we observed an induction of HER2:HER3 heterodimers (Figure 3), suggesting that HER2 allows the kinase-impaired HER3 receptor to gain signaling capacity.

In agreement with the results presented in this manuscript, increased HER3 abundance and ErbB heterodimers after EGFR-targeted therapy has been shown for breast cancer patients [32]. Interestingly, we observed that lapatinib treatment normalized both HER3 phosphorylation and associated HER3:PI3K p85 binding (Figure 4A, Figure 4B and Supplementary Figure S2A). Furthermore, the induction of HER2:HER3 dimers was three times lower upon lapatinib treatment compared to cetuximab (1.7-fold induction vs. 5.4-fold induction) (Figures 4A and 3A). Hence, we suggest that HER2 induces, at least partly, the phosphorylation of cetuximab-induced upregulated HER3, and inhibiting HER2 with lapatinib, coupled with EGFR inhibition, limits this effect.

Due to the cetuximab-induced feedback activation of HER3, we hypothesized that sensitivity to EGFR inhibitors could be improved by inhibiting HER3. Here, we showed that cetuximab combined with lapatinib was able to decrease cell viability upon HER3 knockdown compared to prior to HER3 inhibition (Figure 5B). However, we were not able to elucidate how these events lead to cetuximab resistance in terms of downstream molecules, signaling pathways and biological processes involved. Nevertheless, we showed that combinatorial drug therapy with cetuximab and lapatinib was able to reduce cell viability compared to cetuximab treatment alone (Figure 5B) and it also reverted NRG1-induced resistance to cetuximab in a long-term proliferation assay (Figure 5D). Hence, we believe that this combinatorial drug therapy may be worth to investigate as a potential treatment strategy in order to prevent cetuximab resistance. Interestingly, cetuximab plus lapatinib combination has been assessed in quadruple negative colorectal cancer patient-derived xenografts [42]. In 5 out of 21 cases, this combination caused tumor regression compared to cetuximab treatment alone, supporting the rationale of this drug combination. Remarkably, in 6 out of the 16 cases where cetuximab plus lapatinib was not superior to cetuximab alone, overexpression of the IGF1R ligand IGF2 was found. This is compatible with the RTK array data in our cell lines (Figure 1A) where we do not observe IGF1R phosphorylation induction.

Together, we showed feedback HER3 activation upon cetuximab treatment in *KRAS* and *BRAF* colon cancer cells together with induction of HER2:HER3 heterodimers, which could potentially result in cetuximab resistance in colorectal cancer patients. As a matter of fact, HER3-mediated resistance to EGFR inhibition with gefitinib has been described in lung cancer cells [37]. Interestingly, results from the CALGB 80203 study, where metastatic colorectal cancer patients were randomly allocated to receive either leucovorin/fluorouracil/irinotecan (FOLFIRI) or leucovorin/fluorouracil/oxaliplatin (FOLFOX) with or without cetuximab, showed that *KRAS* wild-type patients with high levels of HER3 did not benefit from cetuximab and were associated with therapy resistance [43]. Overall, our results suggest that after cetuximab treatment not only internalization of EGFR occurs, but also other events might take place, such as feedback activation of HER3 or formation of new ErbB dimers. Dynamic monitoring of changes in dimer formation might be used to predict on treatment increased efficacy of drug combination strategies. The data presented in this manuscript adds to the body of evidence that feedback mechanisms in intermediate cetuximab-sensitve colon cancer cells, which represent most colorectal cancer patients, would dictate the transient and not profound response to cetuximab monotherapy.

## MATERIALS AND METHODS

### Cell lines and reagents

LIM1215, HCA7 and HEK-293T cell lines were cultured in DMEM/F-12 + L-Glutamine + 15 mM HEPES (Gibco) supplemented with 10% fetal bovine serum (FBS) (HyClone) and 1% penicillin/streptomycin (Gibco) at 37°C with 5% CO_2_. LIM1215 cells were obtained from the Ludwig Institute for Cancer Research, New York, NY, USA and HCA7 and HEK-293T cells were obtained from the American Type Culture Collection (ATCC).

Cetuximab (Erbitux^®^) was purchased from Merck Serono, lapatinib (Tykerb/Tyverb^®^) from GlaxoSmithKline, and trastuzumab (Herceptin^®^) and pertuzumab (Perjeta^®^) from Roche. Gefitinib (ZD1839), GDC-0941, trametinib (GSK1120212) and afatinib (BIBW2992) were purchased from Selleck Chemicals. NRG1 was purchased from Peprotech.

### RTK array analysis

Cells were seeded in 10 cm plates (Sarstedt) (3,000,000 cells/plate) and grown 24h before starving them overnight. Cells were treated with cetuximab or control-treated as indicated. The medium was replaced by 10% FBS supplemented medium 10 min before cell harvesting. Cells were lysed, samples prepared and immunoblotting performed according to protocol Proteome Profiler Human Phospho-RTK Array Kit (R&D Systems). Array quantification was performed with ImageJ.

### Western blotting analysis

Cells were seeded in 10 cm plates (Sarstedt) (3,000,000 cells/plate) and treated for different time points in the presence or absence of drugs as indicated. Cells were harvested, washed with ice-cold phosphate buffered saline (PBS) (Gibco) and lysed with NP-40 buffer supplemented with phosphatase inhibitors (Sigma-Aldrich) and protease inhibitor (Roche). Protein concentration was measured by bicinchoninic acid assay (BCA) (Thermo Scientific). Lysates were loaded and separated in sodium dodecyl sulfate (SDS) polyacrylamide gels 4–12% (Life Technologies) and transferred to nitrocellulose membranes (Life Technologies) using iBlot2 Dry Blotting System (Life Technologies). Membranes were blocked with 5% bovine serum albumin (BSA) (Sigma-Aldrich) in tris-buffered saline and Tween 20 (TBS-T) (50 mM Tris, 150 mM NaCl, 0.1% Tween 20, pH 7.6) and incubated overnight at 4°C with the following primary antibodies diluted in 5% BSA in TBS-T: P-HER3 (Y1289) (D1B5) Rabbit mAb, ErbB-3 (C-17) sc-285 Lot #H2712 rabbit polyclonal IgG, P-AKT (Ser473) (193H12) Rabbit mAb, AKT Rabbit Ab, P-ERK1/2 (Thr202/Tyr204) Rabbit Ab, ERK1/2 (137F5) Rabbit mAb, GAPDH (14C10) Rabbit mAb. All primary antibodies were purchased from Cell Signaling and were used in a 1:1000 dilution except for the ErbB-3 (C-17) sc-285 Lot #H2712 rabbit polyclonal IgG antibody that was purchased from Santa Cruz Biotechnology. Membranes were washed with TBS-T, incubated with Goat anti-Rabbit secondary antibody (Dako) 1:5000 in 5% BSA and washed again with TBS-T. Protein signal was detected using chemiluminescent Luminata Forte Western HRP Substrate (Millipore) by exposing Amersham Hyperfilm ECL films (GE Healthccare) to the processor CURIX 60 (AGFA). Western blotting quantification was performed with ImageJ.

### Collaborative Enzyme Enhanced Reactive-immunoassay (CEER)

Cell lysates were prepared according to the manufacturer's protocol and were sent to Prometheus Laboratories Inc. (San Diego, CA, USA) for further analysis.

### Förster resonance energy transfer (FRET)/fluorescence lifetime imaging microscopy (FLIM)

Anti-HER3 (B9A11) was purchased from Monogram Biosciences and anti-HER2 (e2-4001+3B5) was purchased from Thermo Scientific and directly labelled according to the manufacturer's protocol with Alexa546 (X546) and Cy5, respectively.

Imaging and analysis of HER2:HER3 dimer by FRET/FLIM was described previously [44].

### RNA extraction, reverse transcription and quantitative PCR (qPCR)

Cells were seeded in 6-well plates (Cellstar) (400,000 cells/well) and treated in the presence or absence of drugs as indicated. RNA extraction was performed according to protocol MasterPure RNA Purification Kit (Illumina). Reverse transcription was performed with SuperScript II Reverse Transcriptase (Life Technologies) according to the manufacturer's instructions. Gene expression was analyzed using quantitative polymerase chain reaction (qPCR) in 7500 Real Time PCR System (Applied Biosystems). Target gene expression was calculated relative to a reference gene that served as endogenous control (GAPDH) using the comparative Ct method. Each sample was analyzed in duplicate and the mean of all biological replicates was expressed as the result together with the standard deviation. The following primers were used: HER3 forward (5′-GGG GAG TCT TGC CAG GAG-3′), HER3 reverse (5′-CAT TGG GTG TAG AGA GAC TGG AC-3′), NRG1 forward (5′- TGG CTG ACA GCA GGA CTA AC-3′), NRG1 reverse (5′- CTG GCC TGG ATT TCT TC-3′), GAPDH forward (5′-AAG GTG AAG GTC GGA GTC AAC-3′), GAPDH reverse (5′-GAG TTA AAA GCA GCC CTG GTG-3′).

### Myristoylated AKT transfection

Cells were seeded in 6-well plates (Cellstar) (1,000,000 cells/well) and transfected with 2 μg 901 pLNCX-myr-HA-AKT plasmid DNA (Addgene plasmid # 9005) or 2 μg pmaxGFP^®^ vector using the Nucleofector® technology (Lonza). The plasmid encoding myristoylated AKT was provided by William Sellers.

### shHER3 viral transduction

pLKO.1-shHER3 (RHS3979-9630819) and pLKO.1-shGFP were purchased from Open Biosystems. Virus were produced in HEK-293T cells with FuGENE^®^ 6 Transfection Reagent (Promega) and transduced into LIM1215 cells. Infected cells were selected with 2 μg/mL puromycin treatment (Santa Cruz Biotechnology) for 2 days.

### Cell viability analysis

Cells were seeded in 96-well plates (Becton Dickinson) (6,000 cells/well), treated in triplicates in the presence or absence of drugs as indicated and cultured for 72h. Cell viability was measured according to protocol CellTiter-Glo Luminescent Cell Viability Assay (Promega) using the machine VICTOR X3 2030 Multilabel Plate Reader (Perkin Elmer) and expressed as the mean of the triplicates relative to the non-treated control together with the standard deviation.

### Colony formation analysis

Cells were seeded in 24-well plates (Cellstar) (5,000 cells/well), treated in the presence or absence of drugs as indicated and cultured for 10 days refreshing the medium and drugs every two days. Cells were stained with crystal violet solution (Sigma-Aldrich) and washed with PBS.

### Statistical analysis

Unpaired Student's *t*-test was used to compare means of two groups.

## SUPPLEMENTARY FIGURES


