# Supplementary material for: Feedback activation of HER3 attenuates response to EGFR inhibitors in colon cancer cells
Source: Oncotarget. 2016 Dec 9;8(3):4277–88. doi: 10.18632/oncotarget.13834 (PMC5354831; doi:10.18632/oncotarget.13834)
Supplement: Supplementary file 1 [file oncotarget-08-4277-s001.pdf]

Feedback activation of HER3 attenuates response to EGFR inhibitors in colon cancer cells

Supplementary Materials

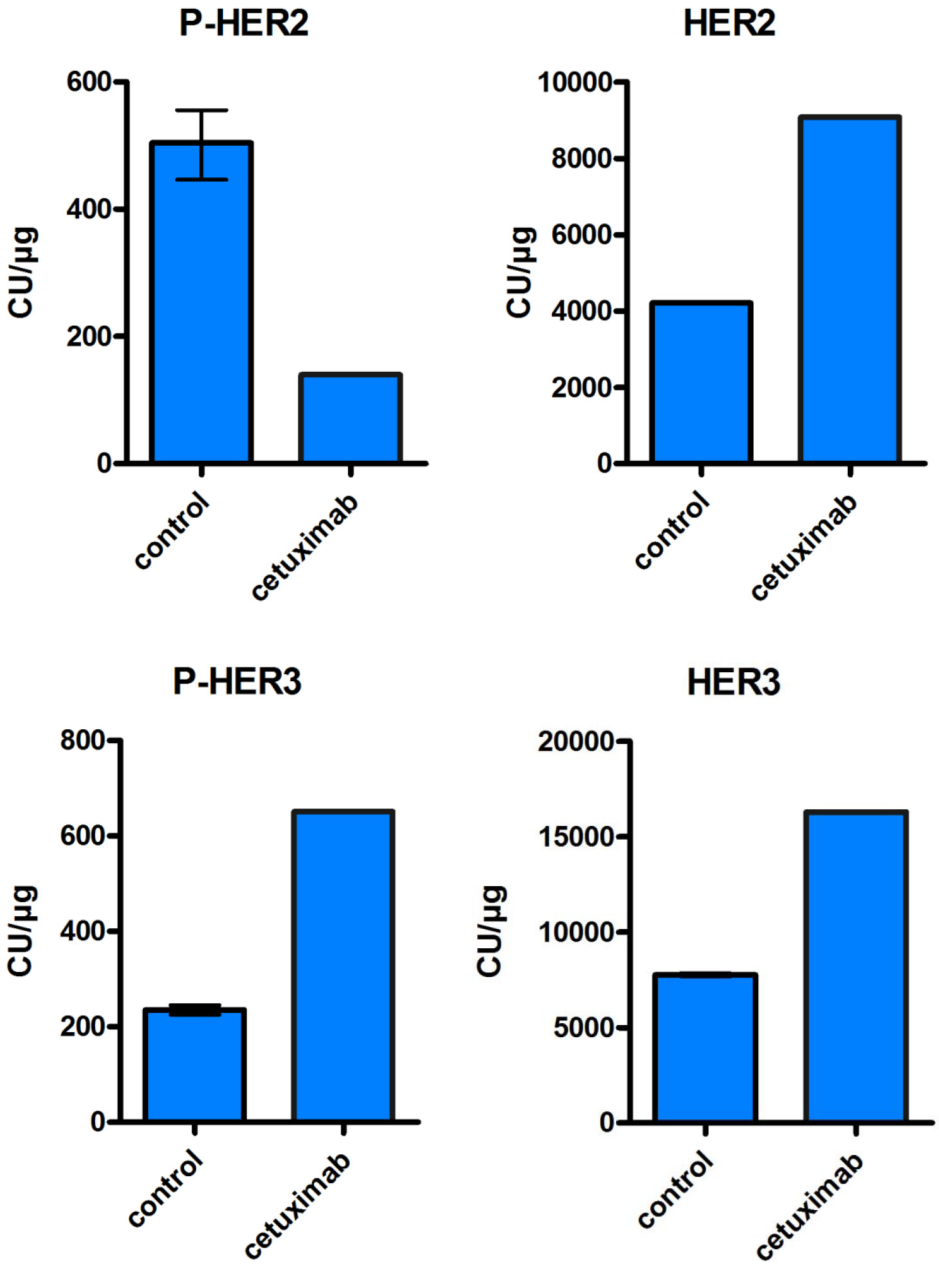

Supplementary Figure S1: Cetuximab treatment results in increased HER3 phosphorylation and total HER2 and HER3 protein levels. (A) LIM1215 cells were treated with cetuximab (50 µg/mL) 24h and both total and phosphorylated levels of HER2 and HER3 were assessed by CEER by Prometheus Laboratories Inc. San Diego, CA (USA). The results are expressed as mean ± SEM. A total of 6 microarray spots were analyzed for each sample. CU: computed unit.

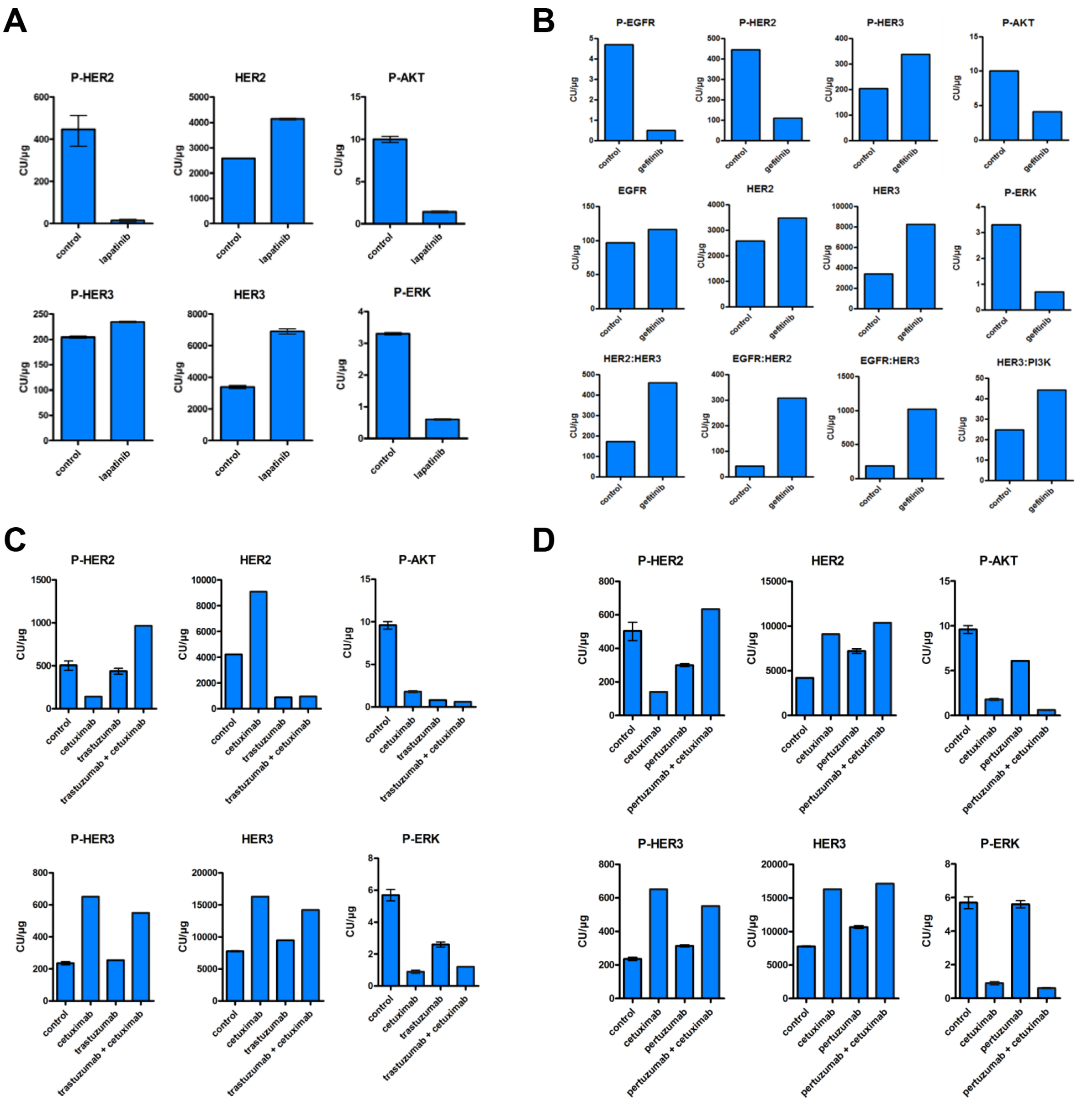

Supplementary Figure S2: Lapatinib normalizes HER3 phosphorylation levels. LIM1215 cells were treated with (**A**) lapatinib (1 µM), (**B**) gefitinib (1 µM), (**C**) cetuximab (50 µg/mL) and trastuzumab (20 µg/mL) or (**D**) cetuximab (50 µg/mL) and pertuzumab (10 µg/mL) for 24h and heterodimer formation between EGFR family members as well as both total and phosphorylated levels of EGFR family members and AKT and ERK phosphorylation levels were assessed by CEER by Prometheus Laboratories Inc. San Diego, CA (USA). The results are expressed as mean ± SEM. A total of 6 microarray spots were analyzed for each sample. CU: computed unit.
